# Exploring Continuous Glucose Monitoring in Gestational Diabetes: A Systematic Review

**DOI:** 10.3390/life15091369

**Published:** 2025-08-28

**Authors:** Bianca-Margareta Salmen, Delia Reurean-Pintilei, Teodor Salmen, Roxana-Elena Bohîlțea

**Affiliations:** 1 Doctoral School, ‘Carol Davila’ University of Medicine and Pharmacy, 050474 Bucharest, Romania; bianca-margareta.mihai@drd.umfcd.ro (B.-M.S.); teodor.salmen@drd.umfcd.ro (T.S.); 2 Department of Medical-Surgical and Complementary Sciences, Faculty of Medicine and Biological Sciences, “Ștefan cel Mare” University, 720229 Suceava, Romania; 3 Department of Diabetes, Nutrition and Metabolic Diseases, Consultmed Medical Centre, 700544 Iasi, Romania; 4 Pitesti Emergency County Hospital, 110283 Pitesti, Romania; 5 Department of Obstetrics and Gynaecology, Filantropia Clinical Hospital, 011132 Bucharest, Romania; roxana.bohiltea@umfcd.ro; 6 Department of Obstetrics and Gynaecology, ‘Carol Davila’ University of Medicine and Pharmacy, 050474 Bucharest, Romania

**Keywords:** gestational diabetes mellitus, GDM, continuous glucose monitoring system, CGMS, pregnancy outcome

## Abstract

(1) Background: Gestational diabetes mellitus (GDM) is a glucose metabolism disorder that typically develops in the second half of pregnancy, transforming a normal pregnancy into a high-risk condition, with both short- and long-term complications for the mother and the fetus. Achieving optimal glycaemic control during pregnancy is essential for preventing these outcomes and could be realized using continuous glucose monitoring systems (CGMSs). This systematic review aims to evaluate the role of the CGMS as a potential diagnostic aid and predictor of maternal and fetal outcomes in GDM. (2) Methods: Following the PRISMA guidelines (protocol ID: CRD42024559169), we performed a literature search using the terms “(continuous glucose monitoring system OR CGMS) AND (gestational diabetes mellitus OR GDM)” in the PubMed, Web of Science, and Scopus databases. (3) Results: Twelve studies were included, all reporting data on CGMS use in pregnancies complicated by GDM. The data included in our analysis are heterogeneous, the results suggesting that the CGMS may offer several advantages such as improved glycaemic control (by avoiding hyper- and hypoglycaemia), better gestational weight management, timely initiation of pharmacologic treatment, lower rates of preeclampsia, and improved neonatal outcomes. (4) Conclusions: the CGMS offers a more detailed assessment of both maternal and fetal exposure to high glucose levels, which could lead to earlier detection of those at risk for GDM complications and better guide treatment regimens, especially timely pharmacological intervention. While the current data are heterogeneous, reporting both limited or no benefits and superior benefits compared to the classic monitoring, larger longitudinal studies are mandatory to validate these findings and to better refine the role of CGMS in the monitoring and management of GDM.

## 1. Introduction

Gestational diabetes mellitus (DM) is a condition that is not diagnosed as overt DM before pregnancy and complicates pregnancy typically in the 2nd or 3rd trimester [1]. The global obesity epidemic affects individuals of reproductive age with a rising prevalence of glucose metabolism disorders during pregnancy, such as type 2 DM (T2DM), alongside type 1 DM (T1DM). The incidence of gestational DM (GDM) is also marked by a significant rise in the incidence [2]. Therefore, GDM presents an increasing trend, suggested also by a study published in 2022 analyzing the prevalence of GDM worldwide, which reports a 14.0% (95% confidence interval (CI) 13.97–14.04%) pooled global standardized prevalence [3].

The effect of GDM can be defined as a major pregnancy complication, as the repercussions of a poor glycaemic control during pregnancy could lead to short-term consequences such as macrosomia [4], the need of instrumental delivery or caesarean section, shoulder dystocia, maternal trauma [5], gestational hypertension, preeclampsia [6], neonatal pathologies such as hypoglycaemia, hyperbilirubinemia, hypocalcaemia, cardiomyopathy, respiratory distress [7,8,9], or even stillbirth [10], not to mention the long-term consequences, which include a 10-fold elevated risk of developing T2DM later in life for the mothers with GDM [11], and a higher risk of becoming overweight [12] or being diagnosed with T2DM in the adulthood for their offsprings [13]. Therefore, an optimum glycaemic control could ensure better pregnancy outcome and diminish fetal and maternal morbidities, as well as provide mothers a basis for lifestyle change in order to minimize the risk of long-term GDM complications.

In the HAPO (Hyperglycaemia and Adverse Pregnancy Outcome) study [14], the investigators found a positive correlation between elevated levels of glycaemia and altered pregnancy outcomes. Identifying early dysglycaemia is of outmost importance, but even if there are multiple glucose control metrics available for metabolic control supervision, the pregnancy state with its characteristics poses some special conditions for some of them. In this direction, the A1c fraction of the haemoglobin (HbA1c) is considered modestly reliable if we take into consideration the physiological changes that occur during pregnancy, respectively, a physiologically lower HbA1c level secondary to the higher red blood cell turnover [15]. Also, for macrosomia, which is best evaluated by postprandial hyperglycaemia, the HbA1c value represents an integrated glucose measurement and is not a reliable marker, so now it is recommended that HbA1c should not be used as a primary or single marker of glycaemic control, nor should it take the place of classic blood glucose monitoring. Continuous glucose monitoring systems (CGMSs) have nowadays become an integral part of DM care, and their utility for managing glycaemic control in pregnancies complicated by T1DM or T2DM is well documented, with current guidelines incorporating these indications. However, further research is needed in order to create a full understanding in terms of the CGMS’s diagnostic capabilities and its prognostic significance for maternal and fetal outcomes, especially considering the multiple nuances of glucose physiology during pregnancy.

Presently, glucose monitoring includes intermittent home self-monitoring blood glucose (SMBG), consisting of at least four blood glucose measurements: fasting, blood glucose, and one hour or two hours after the beginning of each meal [16,17,18]. Glycaemic monitoring also includes non-invasive or minimally invasive methods, respectively, as skin autofluorescence or real-time CGMS [19]. Real-time CGMS, a device introduced in 1999 as an aid in the glycaemic control of patients with DM [20], has evolved nowadays into an indispensable tool in managing glycaemic values, tending towards an ideal control, avoiding hyperglycaemia, and hypoglycaemia [21]. These subcutaneously inserted devices provide information regarding the interstitial fluid glucose levels that the patient experiences during 24 h, providing information on glycaemic fluctuations over the day [20]. Pregnant patients with T1DM, who have been monitored using the CGMS, have had clear benefits, demonstrated in the CONCEPTT (Continuous Glucose Monitoring in Pregnant Women with Type 1 diabetes mellitus) trial: a decrement in both hospital stays length and neonatal intensive care unit (NICU) admissions, a decline in the large for gestational age (LGA) newborns incidence, and a reduction in neonatal hypoglycaemias [22]. Moreover, continuous monitoring of interstitial glucose levels provides a more integrated assessment of both maternal and fetal exposure to high glucose levels, which could lead to earlier detection of those at risk for further complications and could guide timely insulin initiation and more intensive treatment regimens. Given the fact that CGMS technologies are continuously evolving, the quality of life of patients using the CGMS is certainly improved while the benefits of continuous glucose monitoring are maximized, a fact that could support the generalizability of CGMS use in the management of pregnant patients with GDM.

Due to the sparse and varied data on the efficacy of the CGMS in GDM, this systematic review aims to consolidate and present the existing knowledge in this area, respectively, to evaluate the value of CGMSs as a potential diagnostic method and predictor of maternal and fetal outcomes in women with GDM.

## 2. Materials and Methods

According to Preferred Reporting Items for Systematic Reviews and Meta-Analysis Checklist (PRISMA), we created a protocol, registered with number CRD42024559169 in the PROSPERO database. We systematically reviewed the literature, namely the PubMed, Web of Science, and Scopus databases, regarding the CGMS and its association with pregnancy and GDM outcome in comparison, if applicable, with healthy controls.

This review will include original studies, published in English of human patients diagnosed with GDM, monitored using the CGMS and conventional measurement techniques, and that report on GDM screening and diagnosis, glycaemic control, and maternal and neonatal outcomes, including comparisons with healthy controls where applicable. The eligibility criteria used are studies on the population of patients with GDM monitored via the CGMS that report detailed statistical information, including 95% CIs, *p*-values, or correlation levels with various risk factors and disease severity. Exclusion criteria for included studies are letters to the editor, case reports, meeting abstracts, or review articles; redundant publications and studies with unclear or incomplete data.

### 2.1. Research Question and Strategy

We used the “(continuous glucose monitoring system OR CGMS) AND (gestational diabetes mellitus OR GDM)” search criteria in PubMed, Scopus, and Web of Science databases and examined for original articles, respectively, randomized clinical trials, and clinical trials published between January 2015 and December 2024, in the English language, involving human subjects. During the revision process, we enlarged the database search until July 2025.

**Figure 1:**
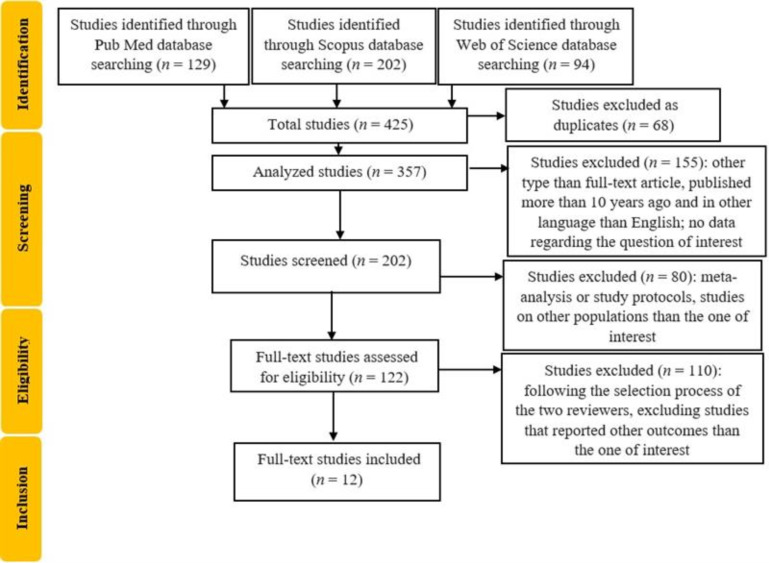
Flowchart of the study selection process.

We identified 202 articles (Figure 1).

### 2.2. Inclusion Criteria

The publication criteria of the studies that were included in this review are (i) original full-text articles, respectively, randomized control trials and clinical trials; (ii) articles published in the last ten years; (iii) articles published in English; and (iv) articles that include human populations with GDM monitored via the CGMS.

### 2.3. Exclusion Criteria

Studies that were excluded from the analysis were (i) literature reviews, meta-analyses, letters to the editors, case reports, or meeting abstracts; (ii) conducted on a non-human population; (iii) conducted on a population under 18 years of age; (iv) studies with unclear or incomplete data. Literature reviews, meta-analyses, and abstracts were excluded from the selection, but, where applicable, they were used for additional references.

### 2.4. Selection of Studies

The selection of the studies was carried out by three reviewers, who excluded from the analysis the articles that were not randomized control trials or clinical trials and that were published in languages other than English. All data were independently recorded by two reviewers in separate databases and only compared at the end of the reviewing process to limit the selection bias. The third reviewer resolved any disagreements that appeared. Each reviewer read the identified papers to ensure that all predefined criteria were met and extracted the following data: title and study details (first author, year of publication, geographic region); study period, sample size and groups, the CGMS model, gestational weeks at sensor mounting and GDM diagnosis method, sensor wearing length, adverse pregnancy outcome, risk estimates, and their respective 95% CIs. Any discrepancies will be resolved through discussions or with input from a third author.

### 2.5. Risk of Bias (Quality) Assessment

The quality of the studies was assessed independently by two reviewers, with the Newcastle–Ottawa Scale (Table 1).

### 2.6. Strategy for Data Synthesis

Our review will include a synthesis centred on the GDM monitoring variants, in order to emphasize whether the CGMS could be a potential diagnostic method and predictor of maternal and fetal outcomes in females with GDM. Due to the anticipated diversity in study design, quality, screening methods, interventions, and outcomes, we plan to perform a narrative synthesis. This approach will involve using text and tables to provide a comprehensive summary and detailed analysis of the characteristics and the results of the studies.

## 3. Results

Table 1 reflects the selection of the studies, using the Newcastle–Ottawa Scale.

**Figure 2:**
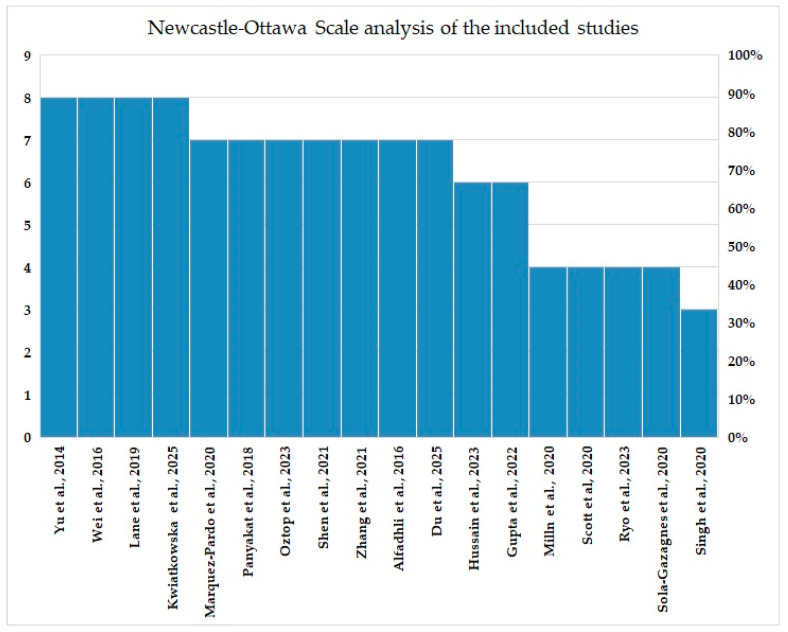
Histogram of the Newcastle–Ottawa Scale analysis of the included studies (Yu et al. [41], Wei et al. [39], Lane et al. [37], Kwiatkowska et al. [23], Marquez-Pardo et al. [32], Panyakat et al. [38], Oztop et al. [27], Shen et al. [30], Zhang et al. [31], Alfadhli et al. [40], Du et al. [24], Hussain et al. [26], Milln et al. [35], Scott et al. [33], Ryo et al. [25], Sola-Gazagnes et al. [34], Gupta et al. [28], Gupta et al. [29], Singh et al. [36]).

We realized a histogram illustration for a better representation of the Newcastle–Ottawa Scale analysis (Figure 2).

The main results from the included studies are summarized in Table 2 and Table 3.

A study published by Marquez-Pardo et al. [32] in 2020 assessed 77 pregnant women with GDM who had been monitored for 6 days using the CGMS. They did not find any association between the rate of overall time above range (TAR) and the materno-fetal outcomes. On the other hand, they concluded that within every percentage point added to the TAR, there was an additional 24% increment in the pharmacological treatment probability. In this regard, the authors predicted using estimated sensitivity (Se) and specificity (Sp) for different TAR cut-off values to identify the patients who were more likely to require pharmacological treatment. For the overall TAR, they obtained a 4% cut-off value (Se 88.8%, Sp 63.7%), 20% before breakfast (Se 77.8%, Sp 67.8%), 18% after breakfast (Se 77.7%, Sp 64.4%), and a cut-off value of 32% before dinner (Se 77.7%, Sp 60.3%), respectively, as well as 6% overnight (Se 88.8%, Sp 62.7%). Their observation implied the fact that patients with GDM presenting an overall TAR higher than 4% should benefit from a particularized follow-up and that CMG could be a very useful tool to detect nighttime hyperglycaemia.

Yu et al. [41] published a study in 2014 on 336 patients with GDM, among whom 147 patients were monitored using the CGMS and SMBG. Their results pointed out that in these patients, the mean amplitude of glycaemic excursions (MAGE) was linked to birth weight (β = 0.196, *p* < 0.001). Also, MAGE was found as an independent risk factor for preeclampsia (OR 3.66, 95% CI 2.16–6.20), as well as for composite neonatal outcome (OR 1.34, 95%CI 1.01–1.77). In the CGMS group, patients undergoing nutritional intervention presented decreased rates of composite neonatal outcome and caesarean section, while those undergoing insulin therapy presented lower birth percentiles and birth weight. The authors entered different values into the analysis. After performing a linear stepwise multiple regression analysis concerning birth weight percentile and birth weight, MAGE2 (MAGE value in the 5th week) was solidly associated with birth weight percentile (β = 0.181, *p* = 0.001, *r*^2^ = 0.133) and birth weight (β = 0.196, *p* = 0.001, *r*^2^ = 0.238). Following a multivariable binary logistic regression, MAGE2 was identified as an independent factor for macrosomia (OR 1.90; 95% CI 1.19–3.04), preeclampsia (OR 3.66; 95% CI 2.16–6.20), neonatal hypoglycaemia (OR, 1.63; 95% CI 1.07–2.48), and also the composite neonatal outcome (OR, 1.34; 95% CI 1.01–1.77). MBG1 (value measured in the 1st week) was identified as an independent risk factor for SGA, LGA, neonatal respiratory distress syndrome (RDS), and also the composite neonatal outcome.

Another prospective study published by Oztop et al. [27] in 2023 on 31 pregnant patients with GDM monitored by the CGMS and SMBG analyzed the outcome and observed that three newborns (9.7%) of mothers who had post-prandial hyperglycaemia (glucose levels > 140 mg/dL) detected by the CGMS had reduced birth weights (2949.9 ± 316.1 g) and also head circumferences (30.3 ± 1.1 cm) in comparison to the other newborns’ birth weights (3042.9 ± 326.2 g) or head circumferences (32.1 ± 1.2 cm), but without a statistical significance (*p* >0.05). Regarding patients with a family history of DM, the infants’ weights and heights were significantly lower (*p* = 0.029 and *p* = 0.043). In addition, they demonstrated that biomarkers such as HbA1c, fructosamine, and 1.5 anhydroglucitol do not reveal the glycaemic variability (observed by the CGMS) in patients with GDM.

In their study published in 2016 on 51 patients with GDM, Wei et al. [39] highlighted that SMBG (adjusted OR 2.40; 95% CI 1.030–5.588; *p* = 0.042), as well as pre-pregnancy body-mass index (BMI) (adjusted OR 0.578; 95% CI 0.419–0.798; *p* = 0.001), were independent risk factors for pregnancy weight gain. In conclusion, early CGMS for GDM mothers reduces gestational weight gain. Although not statistically significant, the caesarean section rate was higher in the control group compared to the CGMS group (69% vs. 60%, *p* = 0.37), and the number of LGA newborns in the CGMS group was reduced (35.3% vs. 52.7% in the CG, *p* = 0.071). In the CGMS group, mothers with the CGMS in the 3rd trimester presented higher MAGE values than those in the 2nd trimester (4.21 ± 0.45 vs. 4.01 ± 0.14, *p* = 0.046). The CGMS group gained less weight (13.56 kg ± 2.81 kg vs. 14.75 kg ± 2.91 kg, *p* = 0.004) compared to SMBG group and there were also a limited number of patients in the CGMS group who gained an inadequate amount of weight (3.9% vs. 5.5%, *p* = 0.039); in addition, the moment of CGMS wearing was important as those who wore the CGMS in the 2nd trimester gained less weight than those in the 3rd trimester (12.72 ± 2.83 kg vs. 14.31 ± 2.64 kg, *p* = 0.003). In contrast, in the CGMS group, insulin was used more than in the SMBG group (31.3% (16 patients) vs. 12.7% (7 patients), *p* = 0.02).

A study published by Shen et al. in 2021 about 97 patients with GDM, monitored using the CGMS [30], reported that with every standard deviation (SD) increment in the nighttime mean glucose level, birth weight percentile was increased by 6.0 (95% CI 0.4, 11.5) points. The same observation was reported about hours per day spent in severe variability (HSSV): each 1 SD increase in HSSV was associated with 6.3 (95% CI 0.4, 12.2) percentage points added to the birth weight percentile.

Lane et al. [37] conducted a prospective study in 2019 on 23 pregnant patients with GDM, divided as follows: 12 patients in the blinded CGMS group (both patients and clinicians involved in the care of the patients were blinded to the CGMS data) and 11 patients in the real-time (RT) CGMS group. They compared the results from the 1st and 4th week of sensor wear between groups. The mean time in range (TIR) in the 1st week for the blinded group was 6497.2 ± 374.1 vs. 7615.3 ± 818.8 min in the RT CGMS group (*p* = 0.3). In the 4th week, the mean TIR was 8398.8 ± 1035.5 in the blinded group vs. 7192.3 ± 2108.1 min in the RT CGMS group (*p* = 0.1). The mean time in hyperglycaemia (TIH) in the 1st week was 459.2 ± 595.6 in the blinded group vs. 508.2 ± 623.8 min in the RT CGMS group (*p* = 0.8). In the 4th week, mean TIH was 278.7 ± 299.6 in the blinded group vs. 633.2 ± 857.1 min in the RT CGMS group (*p* = 0.2). Regarding the neonatal outcome, there were two preterm deliveries in the blinded group, and only one in the RT CGMS group. While the mean birth weight was 3159.2 ± 548.4 g in the blinded group, in the RT CGMS group, the mean birth weight was 3375.5 ± 557.6 g (*p* = 0.4), and there were two LGA newborns in the latter group. There were two SGA newborns in the blinded group and one in the RT CGMS group. In the blinded group, there was one (NICU) admission and one newborn who developed RDS.

Another prospective study was published by Zhang et al. [31] in 2021, regarding 110 pregnant patients with GDM, divided into two groups: 55 patients in the CGMS group and 55 patients in the CG (monitored by SMBG). The groups were similar prior to intervention, in terms of regular blood glucose monitoring, appropriate exercise, weight monitoring, strict diet control, and regular obstetric checkups. After the two weeks of intervention, the patients in the CGMS group presented superior blood glucose monitoring (χ^2^ = 116.042, *p* = 0.000), diet control (χ^2^ = 7.066, *p* = 0.008), weight monitoring (χ^2^ = 9.565, *p* = 0.002), appropriate exercise (χ^2^ = 7.698, *p* = 0.006), and regular obstetric checkups (χ^2^ = 5.492, *p* = 0.019), compared to the CG.

In the study published in 2016 by Alfadhli et al. [40] on 60 pregnant patients with GDM monitored by CGMS and 62 GDM patients monitored by SMBG in the CG, revealed that, although no statistically significant difference was obtained, the AUC for hyperglycaemia and hypoglycaemia were ameliorated in the last day compared to the first day of sensor wearing and the CGMS group necessitated lower doses of insulin (20.67 ± 18.03 vs. 29.83 ± 43.83 in the CG); there were no differences in the number of patients receiving insulin treatment between groups. In the CGMS group, there were lower values regarding the mean fasting plasma glucose (4.71 ± 0.68 vs. 4.99 ± 1.01, *p* = 0.092) and mean postprandial glucose values (5.72 ± 1.59 vs. 6.27 ± 1.36, *p* = 0.057). The CGMS group had a higher rate of PTB (16.3% vs. 9.5%, *p* = 0.373) and an 8.3% rate of premature rupture of membranes compared to 3.9% in the CG. Concerning neonatal outcomes, in the CG, there were 2.4% cases of macrosomia compared to none in the CGMS group. The CGMS group had lower birth weights (2870 ± 610 g vs. 3056 ± 564 g in the CG, *p* = 0.130). The rate of infants with low birth weight was 22.2% in the CGMS group vs. 9.5% in the CG (*p* = 0.147).

## 4. Discussion

Our present study provides an image of the CGMS that is a useful tool to assess both maternal and fetal exposure to high glucose levels, to help detect those at risk for GDM complications earlier, and provide better guided treatment regimens, especially timely pharmacological intervention, and that could be a superior alternative to current screening, diagnosis, and monitoring methods. Because of the heterogeneity of the published studies that met the inclusion criteria, there is a need for larger longitudinal studies, both to validate these findings and to better refine the role of the CGMS in the monitoring and management of GDM.

According to the International Consensus on Time in Range [42], the current recommendations for DM cut-off values in pregnancy have been established: TIR > 70% with a glycaemic target range of 63–140 mg/dL; level 1 time below range (TBR) < 4% with the glycaemic target < 63 mg/dL; and level 2 TBR with less than 1% of the readings being <54 mg/dL. TAR is defined as glycaemic values > 140 mg/dL, and the recommendation is to achieve <25% of readings. Regarding T1DM, the current data reported that a 5–7% increase in TIR in the 2nd half of the pregnancy has been associated with a lower risk of LGA, and improved neonatal outcomes: lower rates of macrosomia, leading to decreased rates of perinatal complications (i.e., shoulder dystocia), diminished rates of neonatal hypoglycaemia, and also lower rates of NICU admissions [43,44].

While the International Consensus has endorsed these values for both GDM and T2DM, based on the studies performed on T1DM, there are standardized measurements for the time spent in each category for T1DM. For instance, for TIR, the goal is to maintain the glycaemic values for more than 16 h and 48 min per day between 63 and 140 mg/dL. Unfortunately, due to the lack of data regarding pregnant women with T2DM or GDM, the International Consensus does not provide cut-off values for the amount of time spent within each category for these patients, and until further recommendations, the glycaemic goals in pregnancy are the same for T1DM, T2DM, and GDM [45]. These are the CGMS and glycaemic parameters.

Maternal blood glucose values were lower in pregnant patients with GDM monitored by CGMS compared to SMBG in three studies: a study by Hussain et al. [26] reported blood glucose values of 89.4 ± 20.1 mg/dL in the CGMS group vs. 102.0 ± 20.5 mg/dL in the SMBG group, Yu et al. [41] reported SDBG lower values in the CGMS group in the last week of sensor wearing compared to the first week (0.8 ± 0.3 vs. 1.3 ± 0.4 mmol/L, *p* < 0.001), and Alfadhli et al. [40] mentioned a decrease in the mean glucose levels (*p* = 0.016), as well as a decrement in the SD of glucose levels (*p* = 0.034) recorded by the CGMS in the last day of sensor wearing. Kwiatkowska et al. [23] also reported a decrease in the MBG in the 2nd and 3rd trimester compared to CG (100.3 ± 9.31 vs. 107.84 ± 9.06 mg/dL, *p* < 0.001 and 100.27 ± 12.63 vs. 109.79 ± 7.73 mg/dL, *p* < 0.001), as well as lower TAR values in the 2nd and 3rd trimester compared to CG (2.86 ± 2.8% vs. 9.13 ± 8.74%, *p* < 0.0013 and 8.49 ± 6.76% vs. 3.22 ± 2.96%, *p* < 0.001). Conversely, Oztop et al. [27] found a positive correlation between mean glucose levels measured by CGMS and SMBG for 72 h (86.1 ± 10.3 vs. 82.9 ± 10.2 mg/dL, r = 0.767, *p* = 0.001). In this direction, the study published by Shen et al. [30] identified a positive correlation between HSSV and fasting plasma glucose (r = 0.39, *p* = 0.0001) and HSSV and the measured two-hour glucose (r = 0.27, *p* = 0.0093).

In the study performed by Yu et al. [41], they observed statistically significant improvements in different parameters of the CGMS over time, more specifically in the 5th week of sensor monitoring concerning SDBG, MAGE, and the mean of daily differences (MODD) (*p* < 0.001). In addition, initiating the CGMS in the 2nd trimester proved its benefits regarding MAGE values compared to the 3rd trimester (4.01 ± 0.14 versus 4.21 ± 0.45, *p* = 0.046), as shown in the article by Wei et al. [39]. The study by Du et al. [24], published in 2025, reported lower fasting blood glucose before delivery in the CGMS group compared to the control group (4.73 ± 1.43 vs. 4.95 ± 1.59 mmol/L, *p* = 0.037). They also compared the first 6 days of CGMS wear with the last 6 days and, despite the fact that they observed a higher MBG in the last 6 days of CGMS compared to the first 6 days, the other parameter values were statistically significantly improved in the last six days: TIR, average daily risk range (ADRR), MAGE, MODD, largest amplitude of glycaemic excursions (LAGE), and blood glucose risk index (BGRI). In addition, another study [23] reported improved MBG and TAR values in the 2nd and 3rd trimester of pregnancy in the CGMS group compared to CG.

Moreover, the CGMS presents the ability to detect different patterns of hyperglycaemia. For example, the study published by Marquez-Pardo [32] reported an increased amount of time spent in hyperglycaemia before dinner, before breakfast, or before lunch.

Most of the findings describe a better glycaemic control while using CGMS, probably due to the fact that CGMS wear aids in increasing responsibility in patients with GDM, contributing to optimizing the diet accordingly to the pregnancy needs and limiting excess eating when consulting the glycaemic values recorded by the system. In consequence, a pregnancy complicated with GDM but with good glycaemic control under diet therapy and exercise and no other complication does not require birth before 39 weeks, and expectant management is recommended until 41 gestational weeks [46].

### 4.1. GMS and Maternal Weight Gain

Regarding weight gain, three studies identified that pregnant women with GDM wearing CGMS gained less weight during pregnancy: Wei et al. [39] concluded in their study that the CGMS group gained less excessive weight compared to the CG (33.3% vs. 56.4%, *p* = 0.039), and also early CGMS monitoring in the 2nd trimester was associated with a lower weight gain compared to the patients monitored in the 3rd trimester (*p* = 0.017). Furthermore, Zhang et al. [31] reported in their work that there were more patients reaching their ideal weight gain in relation to the prepregnancy BMI in the CGMS group compared to the CG (50 vs. 39 cases, *p* = 0.008). The study published by Du et al. [24] reported lower weight gain in the CGMS group (11.8 ± 6.0 vs. 12.9 ± 6.3 kg, *p* < 0.05), and also in the normal weight subgroup monitored by CGMS (9.6 ± 3.2 vs. 10.2 ± 4.5 kg, *p* < 0.05). Therefore, there were improvements in the gestational weight gain among pregnant patients with GDM monitored by CGMS.

Another important aspect is the prepregnancy BMI. Shen et al. [30] found a positive correlation in their study regarding HSSV and prepregnancy BMI (r = 0.25, *p* = 0.0093). Additionally, there are studies in the literature reporting a strong link between prepregnancy BMI and glycaemic metrics. For instance, a study by Liang et al. [47] reported a significant association between TAR and any adverse pregnancy outcome regarding pregnant patients with GDM that were overweight or even obese before pregnancy (OR 1.47, 95% CI 1.18–1.83), not to mention the positive correlation between AUC, daytime MBG, nighttime MBG, or daily MBG, and any adverse pregnancy outcome in these patients compared to underweight or normal weight patients that participated in their study.

CGMS can be a real solution in managing weight gain by optimizing the glycaemic control. Excessive weight gain in pregnancy is detrimental for both mother and fetus. Apart from the increased risk of developing GDM, it promotes weight retention and also obesity for the mother postpartum, with an additional cardiovascular risk in the future [48]; moreover, pregnant patients with excessive weight gain present a higher risk of caesarean delivery OR 1.30 (95% CI 1.25–1.35) [49]. During pregnancy, excessive weight gain is a risk factor for developing gestational hypertension [50] and even preeclampsia in women of all BMI categories, being one the most important causes of preterm birth [51]. Excessive weight gain is associated with LGA and macrosomia, supporting neonate adiposity [52] and childhood obesity [53]. Finally, excessive weight gain is also associated with stillbirth: in a study published by Yao et al. [54] in 2017, obese or morbidly obese pregnant patients who gained excessive weight during pregnancy presented a doubled risk of stillbirth.

Early-life exposures, including high maternal BMI, excessive weight gain during pregnancy, GDM, and pre-existing maternal T2DM, are increasingly recognized as important risk factors for the development of obesity and metabolic dysfunction in the offspring. These maternal conditions may predispose children to altered metabolic programming beginning in utero, creating a lifelong vulnerability to weight-related health issues [55]. One proposed mechanism involves the epigenetic regulation of genes that govern appetite, satiety, and energy homeostasis. Maternal obesity has been shown to influence gene expression in critical brain regions, such as the hypothalamus, that are responsible for regulating food intake and body weight. These changes are believed to be mediated by increased transplacental nutrient transfer, particularly glucose and lipids, which may overstimulate fetal growth pathways and induce long-lasting shifts in neural circuitry and hormonal feedback loops [56]. Such epigenetic modifications can enhance orexigenic signalling, promote fat accumulation, and impair energy expenditure in the offspring, thereby increasing the likelihood of developing obesity and associated cardiometabolic conditions later in life. Importantly, these effects may persist beyond infancy and childhood, contributing to the intergenerational transmission of metabolic disease risk susceptibility [57].

### 4.2. CGMS and Hypoglycaemia

Several studies noticed a statistically significant improvement in the time spent in hypoglycaemia among pregnant patients with GDM while monitored by CGMS. Yu et al. [41] had in their study a rate of 3.4% of patients with hypoglycaemia for more than 30 min in the CGMS group, while in the CG, there was a 19.4% reported rate (*p* < 0.001). Also, in the study conducted by Zhang et al. [31], the hypoglycaemia rate in the CGMS group was 5.45% compared to 21.82% in the CG (*p* = 0.012). Although not statistically significant, other authors observed the same tendency in their studies. For example, Lane et al. [37] noted decreased periods of hypoglycaemia in the RT-CGMS group compared to the blinded group in the first week (*p* = 0.3) and also in the fourth week (*p* = 0.2). Furthermore, Alfadhli et al. [40] underlined an improvement for the AUC for hypoglycaemia and hyperglycaemia in the last day of sensor wearing compared to the first.

The stabilization of glycaemic values throughout pregnancy, with minimizing glycaemic variability and also avoiding extreme values, is critical for the nervous system development of the fetus. Glucose represents the main energy source for brain development. Maternal hypoglycaemia translated into extremely low glycaemic values can compromise oxygen and nutrient delivery to the fetal brain with significant consequences, implying neurological impairment [58]. In this direction, CGMS are useful tools that aid in the better management of the glycaemic control, offering an integrated picture of the glycaemic values, including the nocturnal ones. Regarding the long-term effects, further longitudinal studies are required to determine the long-term consequences on the offspring, and also on the adults that have been exposed in utero to extreme glycaemic variability, as the current data available in the literature is very limited.

### 4.3. CGMS and PT

In the study by Marquez-Pardo et al. [32], the authors highlighted an important finding: a pregnant patient with GDM presents a 24% increase in the probability of PT with every percentage point increase in the TAR value (OR 1.24, 95% CI (1.03–1.45), *p* = 0.006). They also found associations between HP before breakfast and PT (OR 1.04, 95% CI (1.02–1.06), *p* < 0.001), HP after breakfast and PT (OR 1.04, 95% CI (1.01–1.08), *p* = 0.006), HP after dinner and PT (OR 1.02, 95% CI (1.01–1.04), *p* = 0.012), and also HP overnight and PT (OR 1.13, 95% CI (1.05–1.22), *p* = 0.001).

In the study published by Kwiatkowska et al. in 2025 [23], when comparing the patients who received insulin and were monitored by CGMS with those monitored by SMBG, in the CGMS group, the patients were younger, 32 (28.8–34.0) vs. 33.0 (30.0–37.0), *p* = 0.035. They had their first visit earlier in pregnancy, at, respectively, 19.0 (11.8–26.3) GW vs. 26 (15–29) in the CG, *p* = 0.004, and they received an earlier diagnosis compared to CG, at 11.0 GW(8.0–23.0) compared to 24.0 (10.0–25.0) GW, *p* < 0.001. The patients in the CGMS and insulin group also presented lower prepregnancy BMI: 24.3 (21.9–29.0) compared to 26.0 (22.9–30.1) kg/m2 in the CG, *p* = 0.048, and they also had more visits compared to CG: 8 (5–9) vs. 5 (4–8), *p* < 0.001. Furthermore, the authors [23] reported a higher number of insulin-treated patients among those monitored by CGMS compared to the CG, respectively, 98.1% compared to 81.3%, *p* = 0.005, and they received earlier basal insulin therapy compared to the CG, respectively, at 15 (11.5–27) vs. 27 (16–30) GW, *p* < 0.001. Another study [39] recorded a higher rate of insulin necessity in the CGMS group compared to CG (31.3% vs. 12.7%, *p* = 0.02). This finding was also observed in the literature data: a study by Kestilä et al. [59], on 36 pregnant patients with GDM monitored by CGMS and 37 patients monitored by SMBG, noticed a higher rate of antihyperglycemic drug therapy (insulin, metformin, or both) in the CGMS group compared to the CG (31% vs. 8%, *p* = 0.0149).

In contrast, the study by Alfadhli et al. [40] observed no difference between groups regarding the number of patients receiving treatment, but they observed that in the CGMS group, there were lower doses of insulin required (20.67 ± 18.3 vs. 29.83 ± 43.83 in the CG).

These observations are very important for both the clinician and the patient. Apart from the glycaemic values recorded during the course of the day, a major advantage of CGMS is represented by the overnight glycaemic recordings, as nocturnal hyperglycaemia can be identified more easily, and subsequent intervention, namely pharmacotherapy, can begin timely. Another notable observation is the reduced insulin requirement in patients monitored using CGMS, compared to those using SMBG, potentially attributable to increased patient awareness facilitated by the real-time feedback provided by CGMS.

### 4.4. CGMS and Associated Complications

In their study, Panyakat et al. [38] reported no correlation between maternal glycaemic parameters and gestational hypertension. Gestational hypertension was encountered in three cases of blinded CGMS compared to 1 in the RT-CGMS group (*p* = 0.6) in the study published by Lane et al. [37]. The same study group reported two cases of preeclampsia in the blinded CGMS group and one in the RT-CGMS group (*p* = 1). Statistical significance was obtained in the study published by Yu et al. [41], in which pregnant patients monitored by CGMS presented lower rates of preeclampsia compared to the CG (3.4% vs. 10.1%, *p* = 0.019); furthermore, they identified MAGE as an independent risk factor for preeclampsia (OR 3.66, 95% CI 2.16–6.20).

Regarding the rate of caesarean section, a study by Panyakat et al. [38] reported no correlation between glycaemic parameters and the caesarean section rate. In contrast, there were two studies with similar conclusions: Wei et al. [39] reported a lower rate of caesarean section in the CGMS group compared to the CG (60% vs. 69%, *p* = 0.37), and Yu et al. [41] found a statistically significant decrease primary caesarean section rate in the CGMS group (34.7% vs. 46.6%, *p* = 0.028).

Finally, CGMS monitoring could capacitate pregnant women with GDM to be more responsible or raise awareness concerning the prevention of adverse pregnancy outcomes: in the study by Zhang et al. [31] the patients in the CGMS group presented superior blood glucose monitoring (*p* = 0.000), diet control (*p* = 0.008), weight monitoring (*p* = 0.002), appropriate exercise (*p* = 0.006), and regular obstetric checkups (*p* = 0.019).

### 4.5. CGMS and Fetal/Neonatal Weight

Regarding fetal/neonatal weight, the results are heterogeneous. A study reported no statistically significant association between glycaemic parameters and LGA or birth weight (*p* > 0.05) [38]. Another study by Lane et al. [37] reported two cases of fetal macrosomia in the RT-CGMS group compared to none in the blinded CGMS group (*p* = 0.2), and two cases of SGA in the blinded group and one in the CGMS group. Conversely, in their work, Oztop et al. [27] reported a negative correlation between AUC > 140 mg/dL and mean birth weight (r = −0.428, *p* = 0.016), and between MAD% and the mean head circumference of the newborns (r = −0.459, *p* = 0.009). Furthermore, in the study conducted by Marquez-Pardo et al. [32], the authors reported a statistically significant association between TIH after lunch and macrosomia (OR 1.04, 95% CI (1.01–1.08), *p* = 0.035) and also between TIH and LGA infants (OR 1.05, 95% CI (1.01–1.09), *p* = 0.010). In the same direction, in the study published by Yu et al. [41], the CGMS group presented lower mean infant birth weight (3138 ± 484 g vs. 3345 ± 508 g, *p* < 0.001), with lower mean birth weight percentile (66 (35–82) vs. 82 (64–91), *p* < 0.001), fewer LGA fetuses (20 vs. 48 in the CG, *p* = 0.025), and a lower rate of macrosomia (4.1% vs. 10.8% in the CG, *p* = 0.01). Their analysis also determined that MAGE in the 5th week was strongly associated with birth weight percentile (β = 0.181, *p* = 0.001, *r*^2^ = 0.133) and birth weight (β = 0.196, *p* = 0.001, *r*^2^ = 0.238), also an independent factor for macrosomia (OR 1.90; 95% CI 1.19–3.04); also, MBG in the 1st week was determined as an independent factor for SGA and LGA [41]. Although not statistically significant, the study performed by Shen et al. [30] reported that 1 SD increase in the HSSV (3.91 h) determined a 6.3 (95% CI (0.4–12.2)) increase in the birth weight percentile, and also each 1 SD increase in the mean night-time glycemia (0.53 mmol/L) increases birth weight percentile with 6.0 (95% CI (0.4–11.5)). The latter findings are in accordance with the data from a prospective study by Liang et al. [47] published in 2023, on 1302 pregnant patients with GDM monitored by CGMS, which reported that higher levels of several glycaemic parameters were associated with an increased risk of having a LGA fetus: daily MBG (OR 1.60; 95% CI 1.35–1.91), night-time MBG (OR 1.54; 95% CI 1.30–1.83), day-time MBG (OR 1.51; 95% CI 1.27–1.79), TIR (per 1 SD, OR 1.30; 95% CI 1.05–1.61), TAR (OR 1.36; 95% CI 1.19–1.56), AUC (OR 1.60; 95% CI 1.35–1.90), and MAGE (OR 1.20; 95% CI 1.01–1.42). Their study also found a correlation between per 1 SD modification in TBR and a lower risk of LGA (OR 0.62, 95% CI (0.48–0.80)).

Fetal weight is a good indicator of glycaemic control. Maternal hyperglycaemia is responsible for the increase in fetal weight [14]. The fact that the conclusions of the studies included in our analysis are heterogeneous and most of the results are not statistically significant is probably due to the limited number of patients included in the studies. When analyzing larger studies [32,41,47], increased values of the CGMS parameters (daily MBG, TAR, night-time MBG, MAGE) are strongly associated with higher risks of LGA fetuses or macrosomia. Simultaneously, one larger study [26] reported lower rates of macrosomia in patients monitored by CGMS, being an important argument for the use of CGMS in patients with GDM as macrosomia is linked to shoulder dystocia, failure of induced labour, and an increased risk of caesarean delivery [60].

### 4.6. CGMS and Fetal/Neonatal Complications

A study found no correlation between maternal glycaemic parameters and PTL, PTB, neonatal hypoglycaemia, RDS, or NICU admissions [38]. PTB presented a higher rate in the CGMS group (16.3% vs. 9.5% in the CG, *p* = 0.373), with 8.3% of cases being due to the premature rupture of membranes in the study conducted by Alfadhli et al. [40]. On the opposite pole, Yu et al. [41] reported a reduction in the number of premature deliveries in the CGMS group (4.8% vs. 11.8% in the CG, *p* = 0.024). The same study group noted statistically significant improvements in the CGMS group concerning the cases of neonatal hypoglycaemia (5.5% vs. 14% in the CG, *p* = 0.011), neonatal hyperbilirubinemia (2.7% vs. 9.7%, *p* = 0.012), RDS (1.4% vs. 5.9%, *p* = 0.034), and composite neonatal outcome (27.4% vs. 49.5%, *p* < 0.001) [41]. The same authors concluded that MAGE in the 5th week was identified as an independent factor for neonatal hypoglycaemia (OR, 1.63; 95% CI 1.07–2.48) and composite neonatal outcome (OR, 1.34; 95% CI 1.01–1.77), while MBG in the 1st week was identified as an independent risk factor for neonatal RDS and composite neonatal outcome. Du et al. [24] reported a lower rate in the CGMS group compared to the CG concerning neonatal hypoglycaemia (7.7% vs. 12.6%, *p* < 0.05), neonatal hyperbilirubinemia (16.5% vs. 21.6%, *p* < 0.05), and NICU admissions (49.6% vs. 64.5%, *p* < 0.05).

While Lane et al. [37] reported one NICU admission and one infant who developed RDS in the blinded CGMS group compared to none in the RT-CGMS group, the data in the literature reported that pregnant patients with GDM and higher levels of TAR present a higher risk of NICU admission (per 1 SD the OR was 1.24, 95% CI 1.07–1.44); TAR ≥5% was linked to an increased risk of premature delivery (OR 2.17, 95% CI 1.11–4.24) [47]. The same group identified MBG as a critical glycaemic parameter: every 5 mg/dL rise in daily MBG determined a 12% (95% CI 1.05–1.19) more elevated risk of any adverse pregnancy outcome, and also a 30% (95% CI 1.18–1.44) greater risk of LGA; in addition, increased values of daytime MBG were linked to an increased risk of the premature rupture of membranes (OR 1.46, 95% CI 1.03–2.05) [47]. Another study by Fishel Bartal et al. [61], published in 2023, concerned 92 pregnant patients undergoing GDM screening, with diagnosed pregnant patients with GDM, revealing that a TAR ≥ 10% was statistically significantly associated with composite adverse neonatal outcome compared to the patients with TAR < 10% (63% vs. 18%, *p* = 0.001), hypoglycaemia (47% vs. 14.5%, *p* = 0.009), and a longer length of stay (4 vs. 2 days, *p* = 0.03).

## 5. Other Findings

Independent of the CGMS parameters and materno-fetal outcomes, there were studies that communicated other associations. Firstly, an interesting observation stated by Oztop et al. [27] is that the biomarkers HbA1c, fructosamine, and 1.5 anhydroglucitol do not reflect the glycaemic variability in patients with GDM, a variability objectified by CGMS. Secondly, Zhang et al. [31] proved the fact that CGMS has a superior acceptance by pregnant patients with GDM, achieving a compliance rate of 94.55% in the CGMS group compared to 74.55% in the CG (*p* = 0.004). Panyakat et al. [38] observed positive correlations between gestational weight gain and birth weight percentiles (r = 0.437, *p* = 0.002), and also between maternal height and birth weight percentiles (r = 0.369, *p* = 0.011).

**Figure 3:**
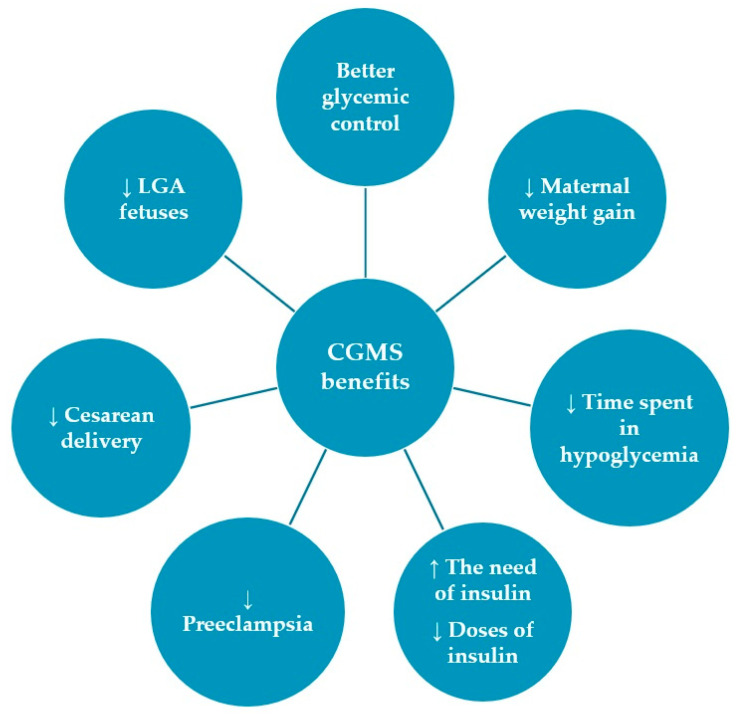
Summary of the main benefits of CGMS in GDM (↑—increase, ↓—decrease).

For a better representation of the main benefits of monitoring GDM via CGMS, we created an illustration that summarizes the essential findings of our study (Figure 3).

### 5.1. Future Directions

At present, the use of CGMS has been standardized among pregnant patients with T1DM [45,62], but there is insufficient data to support the global use of CGMS in GDM or T2DM, monitoring for these last two types of DM being performed via SMBG. There is a real need to establish the advantages and limitations of CGMS in GDM, and further studies are mandatory to investigate GDM-specific cut-offs for the CGMS parameters (TIR, TAR, TBR, etc.). Monitoring GDM via CGMS could be a valuable step forward into enlightening the association between nocturnal hyperglycaemia or hyperglycaemic patterns and pregnancy outcomes, as well as timely pharmacological treatment and materno-fetal outcome. There is a clear requirement for further larger studies, with similar designs, similar devices used, homogeneity in glycaemic cut-offs, measurements, and terminology, exploring long-term neonatal outcomes along with the financial impact on the healthcare system. Future studies could include non-human populations or even adolescents, as they are a socially vulnerable category that requires close monitoring.

### 5.2. Strengths and Limitations

The main strength of our study is represented by the comprehensive analysis reflected by various methods of patient evaluation, different types of evaluated outcomes, and by the diverse methodology for each included study. In contrast, an important limitation is represented by the limited sample sizes of the included studies, followed by the GDM diagnosis discrepancy, the absence of a cost effectiveness analysis of CGMS compared to SMBG, the lack of homogeneity regarding CGM metrics, and the study design and devices used being heterogeneous, especially when analyzing the type of sensor, the period of the sensor wearing length (from 48 h to months), and the gestational weeks at sensor mounting, including the selected population, and the reported adverse outcomes. Moreover, regarding CGMS and GDM, the possible biases are of publication, due to the fact that more frequently studies with positive outcomes or correlations are published, and of language, due to the exclusion of non-English studies. Another limitation might be the fact that the study’s interpretation could be influenced by the experience level of the authors with the Newcastle–Ottawa Scale, which is, in fact, very accessible.

## 6. Conclusions

Continuous monitoring of interstitial glucose levels offers a more detailed assessment of both maternal and fetal exposure to high glucose levels, which could lead to earlier detection of those at risk for GDM complications and better guide treatment regimens. This approach might also provide a superior alternative to current screening, diagnosis, and monitoring methods, and also broaden the use of CGMS in other types of DM in pregnancy. Given the significant risks associated with poorly managed hyperglycaemia during pregnancy, non-invasive monitoring via CGMS has the potential to improve glycaemic control and enhance outcomes for both mother and fetus.

## Figures and Tables

**Table 1:** Newcastle–Ottawa Scale analysis of the included articles.

Author (Reference)	Selection				Comparability	Outcome			Total Score	Quality
	Representativeness of the Exposed Cohort	Selection of the Non-Exposed Cohort	Ascertainment of Exposure	Demonstration That Outcome of Interest Was Not Present at Start of Study	Comparability of Cohorts Based on the Design or Analysis	Assessment of Outcome	Was Follow-Up Long Enough for Outcomes to Occur	Adequacy of Follow-Up of Cohorts		
Kwiatkowska et al. [23], 2025	*	*	*	*	*	*	*	*	8	Good
Du et al. [24], 2025	*	*	*	*	*	*	*	-	7	Good
Ryo et al. [25], 2023	-	-	-	-	*	*	*	*	4	Poor
Hussain et al. [26], 2023	*	-	*	*	*	*	*	-	6	Good
Oztop et al. [27], 2023	*	*	*	*	*	*	*	-	7	Good
Gupta et al. [28], 2022	-	-	-	-	-	*	*	*	3	Poor
Gupta et al. [29], 2022	-	-	-	-	-	*	*	*	3	Poor
Shen et al. [30], 2021	*	*	*	*	*	*	*	-	7	Good
Zhang et al. [31], 2021	*	*	*	*	*	*	*	-	7	Good
Marquez-Pardo et al. [32], 2020	*	*	*	*	-	*	*	*	7	Good
Scott et al. [33], 2020	-	-	-	-	*	*	*	*	4	Poor
Sola-Gazagnes et al. [34], 2020	*	-	-	-	*	-	*	*	4	Poor
Milln et al. [35], 2020	*	*	*	*	*	-	-	-	4	Poor
Singh et al. [36], 2020	-	-	-	-	*	-	*	*	3	Poor
Lane et al. [37], 2019	*	*	*	*	*	*	*	*	8	Good
Panyakat et al. [38], 2018	*	*	-	*	*	*	*	*	7	Good
Wei et al. [39], 2016	*	*	*	*	*	*	*	*	8	Good
Alfadhli et al. [40], 2016	*	*	*	*	*	*	-	*	7	Good
Yu et al. [41], 2014	*	*	*	*	*	*	*	*	8	Good

“*” indicates that the article meets the criteria mentioned above; “-” indicates that the article does not meet the abovementioned criteria.

**Table 2:** CGMS intervention characteristics of the included studies.

First Author, Publication Year	Country	Sample Size; Groups	Study Period	CGMS Model	GW at Sensor Mounting; GDM Diagnosis Method	Sensor Wearing Length
Kwiatkowska [23], 2025	Poland	277 GDM: 53 CGMS, 224 CG SMBG	January 2023–June 2023	Abbot Free-Style Libre (Abbott Park, IL, USA.)	24–28 GW; 75 g glucose OGTT	From diagnosis until delivery
Du [24], 2025	China	3062 GDM: 466 CGMS, 2596 CG SMBG	November 2021–October 2022	Abbot Free-Style Libre	24–28 GW; 75 g glucose OGTT	14 days
Hussain [26], 2023	United States	41 GDM	NS	Abbot Free-Style Libre	NS	14 days
Oztop [27], 2023	Turkey	31 GDM (CGMS and SMBG)	NS	Medtronic Enlite Glucose Sensor^®^, (Galway, Ireland)	35 GW, GDM diagnosis was according to the Turkish Society of Endocrinology and Metabolism Diabetes Mellitus guidelines	72 h
Shen [30], 2021	China	97 CGM	January 2017–November 2018	Abbott Freestyle Libre	22–28 GW, 75 g glucose OGTT	5–14 days
Zhang [31], 2021	China	55 CGM 55 CG (SMBG)	April 2019–April 2020	Abbott Diabetes Care Ltd.	24–28 GW, NS	2 weeks
Marquez-Pardo [32], 2020	Spain	77 GDM	February 2016–July 2018	Medtronic iPro™ 2 CGMS	30.2 ± 2.16; 50 g glucose OGTT load test followed by 100 g glucose OGTT diagnosis test	6 days
Lane [37], 2019	United States	12 CGMS blinded * 11 RT CGMS	December 2017–May 2018	Medtronic iPro 2	24–27^+6^ GW; 50 g glucose test ≥ 135 mg/dL, followed by 100 g glucose OGTT or 50 g glucose test ≥ 200 mg/dL	4 weeks
Panyakat [38], 2018	Thailand	47 GDM	1 August–31 December 2016	iPro2^®^ CGMS	28–32 GW; 50 g glucose load test, followed by 100 g glucose OGTT	At least 72 h; 85.5 ± 12.83 h
Wei [39], 2016	China	51 CGM: early subgroup (2nd trimester) 24, latter subgroup (3rd trimester) 27; 55 SMBG	September 2011–December 2012	Gold Medtronic MiniMed	24–28 GW, 75 g glucose OGTT	48–72 h
Alfadhli [40], 2016	Saudi Arabia	60 CGM 62 SMBG	October 2011–June 2014	Guardian^®^ RT CGMS (Medtronic MiniMed)	26 ± 5 GW; IADPSG criteria	3–7 days within 2 weeks after the GDM diagnosis (mean duration 66.8 ± 2.3 h)
Yu [41], 2014	China	336 GDM; 189 SMBG (7 times/day)—CG and 147 CGMS and SMBG	April 2011–August 2012	Medtronic Minimed	24–28 GW; 75 g glucose OGTT	72 h per week, two consecutive weeks, after that, 72 h each 2 to 4 weeks; in the fifth week all patients underwent CGMS for 72 h

GDM—gestational diabetes mellitus; GW—gestational weeks; CGMS—continuous glucose monitoring system, DM—diabetes mellitus, NS—not specified; SMBG—self-monitoring blood glucose; CG—control group; OGTT—oral glucose tolerance test; * blinded—both patients and clinicians involved in the care of the patients blinded to the CGMS data; IADPSG—International Association of Diabetes in Pregnancy Study Groups.

**Table 3:** Clinical outcomes of the included studies.

First Author, Publication Year	Adverse Pregnancy Outcome	Outcome	*p* Value
Kwiatkowska [23], 2025	Lower MBG in the 2nd and 3rd trimester compared to CG	100.3 ± 9.31 vs. 107.84 ± 9.06 mg/dL and 100.27 ± 12.63 vs. 109.79 ± 7.73	*p* < 0.001 and *p* < 0.001
	Lower TAR in the 2nd and 3rd trimester compared to CG	2.86 ± 2.8% vs. 9.13 ± 8.74% and 8.49 ± 6.76% vs. 3.22 ± 2.96%	*p* < 0.0013 and *p* < 0.001
	Earlier basal insulin therapy compared to CG	15 (11.5–27) vs. 27 (16–30) GW	*p* < 0.001
	Higher number of insulin treated patients compared to CG	98.1% vs. 81.3%	*p* = 0.005
	Newborns with bigger body length compared to CG	54 (53–56) vs. 53 (51–55) cm	*p* = 0.005
Du [24], 2025	Neonatal hypoglycaemia	7.7% vs. 12.6% in the CG	*p* < 0.05
	Neonatal hyperbilirubinemia	16.5% vs. 21.6% in the CG	*p* < 0.05
	NICU admission	49.6% vs. 64.5% in the CG	*p* < 0.05
	Lower WG	11.8 ± 6.0 vs. 12.9 ± 6.3 kg in the CG	*p* < 0.05
	Lower WG in the normal weight subgroup	9.6 ± 3.2 vs. 10.2 ± 4.5 kg in the CG	*p* < 0.05
	Lower fasting blood glucose before delivery	4.73 ± 1.43 vs. 4.95 ± 1.59 mmol/L in the CG	*p* = 0.037
	Better TIR in the last 6 days of CGMS compared to the first 6 days of CGMS	97.0 (86.8–99.3) vs. 84.75 (68.9–9.6) %	*p* < 0.001
	Higher MBG in the last 6 days of CGMS compared to the first 6 days of CGMS	5.35 ± 0.83 vs. 5.06 ± 1.02 mmol/L	*p* < 0.001
	Better glucose management indicators in the last 6 days of CGMS compared to the first 6 days of CGMS	36.87 ± 4.63 vs. 37.98 ± 3.9 mmol/L	*p* = 0.001
	Lower ADRR in the last 6 days of CGMS compared to the first 6 days of CGMS	12.57 ± 4.54 vs. 18.8 ± 6.86 mmol/L	*p* > 0.001
	Lower MAGE in the last 6 days of CGMS compared to the first 6 days of CGMS	2.8 ± 0.79 vs. 2.95 ± 0.84	*p* = 0.013
	Lower MODD in the last 6 days of CGMS compared to the first 6 days of CGMS	0.84 ± 0.28 vs. 0.9 ± 0.28	*p* = 0.002
	Lower LAGE in the last 6 days of CGMS compared to the first 6 days of CGMS	5.99 ± 1.59 vs. 6.72 ± 1.5	*p* < 0.001
	Lower BGRI in the last 6 days of CGMS compared to the first 6 days of CGMS	2.73 (1.81–3.87) vs. 4.39 (2.73–6.47)	*p* < 0.001
Hussain [26], 2023	NS	Mean glucose values: capillary blood glucose—102.0 ± 20.5 and intermittently scanned continuous glucose monitor 89.4 ± 20.1 mg/dL	NS
Oztop [27], 2023	Mean glucose level (CGMS vs. SMBG six times a day for 72 h)	86.1 ± 10.3 mg/dL vs. 82.9 ± 10.2 mg/dL; r = 0.767	*p* < 0.001
	AUC >140 mg/dL and mean birth weight	r = −0.428	*p* = 0.016
	MAD% and babies’ mean head circumference	r = −0.459	*p* = 0.009
Shen [30], 2021	HSSV was found positively associated with fasting glucose	r = 0.39	*p* = 0.0001
	HSSV and 2 h glucose	r = 0.27	*p* = 0.0093
	HSSV and prepregnancy BMI	r = 0.25	*p* = 0.0118
	Each 1 SD (3.91 h) elevation in HSSV increases birth weight percentile	6.3 (95% CI 0.4, 12.2)	NS
	Each 1 SD (0.53 mmol/L) elevation in mean nighttime glycemia level increases birth weight percentile	6.0 (95% CI 0.4, 11.5)	NS
Zhang [31], 2021	Hypoglycaemia CGMS group vs. CG	3 cases (5.45%) vs. 12 cases (21.82%)	*p* = 0.012
	WG (ideal WG in relation to prepregnancy BMI): CGMS group vs. CG	50 cases (90.91%) vs. 39 cases (70.91%)	*p* = 0.008
	Compliance rate for CGMS group vs. CG	94.55% vs. 74.55%	*p* = 0.004
Marquez-Pardo [32], 2020	TIH after lunch and macrosomia	OR 1.04, 95% CI (1.01–1.08)	*p* = 0.035
	TIH and LGA infants	OR 1.05, 95% CI (1.01–1.09)	*p* = 0.010
	PT and TAR	OR 1.24, 95% CI (1.06–1.45)	*p* = 0.006
	PT and HP before breakfast	OR 1.04 95% CI (1.02–1.06)	*p* < 0.001
	PT and HP after breakfast	OR 1.04 95% CI (1.01–1.08)	*p* = 0.006
	PT and HP after dinner	OR 1.02 95% CI (1.01–1.04)	*p* = 0.012
	PT and HP over night	OR 1.13 95% CI (1.05–1.22)	*p* = 0.001
Lane [37], 2019	Shorter periods in hypoglycaemia in the RT group vs. blinded in the 1st week, respectively, in the 4th week	532.9 ± 581.6 vs. 1410.5 ± 2871 min, respectively, 274.5 ± 317.4 vs. 492.4 ± 409	*p* = 0.3, respectively, *p* = 0.2
	Gestational hypertension blinded group vs. RT CGMS	3 cases (25%) vs. 1 case (9.1%)	*p* = 0.6
	Preeclampsia blinded group vs. RT CGM	2 cases (16.7%) vs. 1 case (9.1%)	*p* = 1
	Fetal macrosomia blinded group vs. RT CGM	0 vs. 2 cases (18.2%)	*p* = 0.2
Panyakat [38], 2018	LGA, birth weight, primary caesarean section, cephalopelvic disproportion, PIH, PPH, PTL, PTB, neonatal hypoglycaemia, RDS, NICU admission	No correlation found between glycaemic parameters and pregnancy outcomes	*p* > 0.05
	Gestational WG and birth weight percentiles	*r* = 0.437	*p* = 0.002
	Maternal height and birth weight percentiles	*r* = 0.369	*p* = 0.011
Wei [39], 2016	Lower excessive WG vs. CG	33.3% vs. 56.4%	*p* = 0.039
	Initiated CGMS earlier gained less weight		*p* = 0.017
Alfadhli [40], 2016	Decrease in the mean sensor glucose, respectively, SD of sensor glucose in the CGMS group in the last day of sensor wearing	-	*p* = 0.016, respectively, *p* = 0.034
Yu [41], 2014	SD of blood glucose; MAGE; and MODD in the CGMS group in the 5th week compared to the first week	0.8 ± 0.3 vs. 1.3 ± 0.4 mmol/L; 1.8 ± 0.6 vs. 3.0 ± 1.1 mmol/L; 1.0 ± 0.2 vs. 1.4 ± 0.4	*p* < 0.001; *p* < 0.001; *p* < 0.001
	Hypoglycaemia > 30 min/day in the CGMS group vs. CG	3.4% vs. 19.4%	*p* < 0.001
	Preeclampsia	5 cases (3.4%) vs. 19 cases (10.1%) in the CG	*p* = 0.019
	Primary caesarean delivery	51 cases (34.7%) vs. 88 cases (46.6%) in the CG	*p* = 0.028
	Mean infant birth weight	3138 ± 484 g vs. 3345 ± 508 g in the CG	*p* < 0.001
	Premature delivery	7 cases (4.8%) vs. 22 cases (11.8%) in the CG	*p* = 0.024
	Birth weight percentile	66 (35–82) vs. 82 (64–91) in the CG	*p* < 0.001
	Macrosomia	6 (4.1%) vs. 20 (10.8%) in the CG	*p* = 0.025
	LGA	20 (13.7%) vs. 48 (25.8%) in the CG	*p* = 0.01
	Neonatal hypoglycaemia	8 (5.5%) vs. 26 (14%) in the CG	*p* = 0.011
	Neonatal hyperbilirubinemia	4 (2.7%) vs. 18 (9.7%) in the CG	*p* = 0.012
	Respiratory distress syndrome	2 (1.4%) vs. 11 (5.9%) in the CG	*p* = 0.034
	Composite neonatal outcome	40 (27.4%) vs. 92 (49.5%) in the CG	*p* < 0.001

CGMS—continuous glucose monitoring system; DM—diabetes mellitus, NS—not specified; ADRR—average daily risk range; LAGE—largest amplitude of glycaemic excursions; BGRI—blood glucose risk index; TIH—time in hyperglycaemia; LGA—large for gestational age; PT—pharmacological treatment; SD—standard deviation; TAR—time above range; HP—hyperglycaemia patterns; SMBG—self-monitoring blood glucose; CGMS—continuous glucose monitoring system; CG—control group; MBG—mean blood glucose, SDBG—SD of blood glucose, MAGE—mean amplitude of glycaemic excursions; MODD—mean of daily differences, RT-CGMS—real time CGMS; PIH—pregnancy induced hypertension; PPH—postpartum haemorrhage; PTL—preterm labour; PTB—preterm birth; RDS—respiratory distress syndrome; NICU—neonatal intensive care unit; AUC—area under the curve; MAD%—percentage of mean absolute differences; HSSV—hours per-day spent in severe variability; BMI—body mass index; blinded—both patients and clinicians involved in the care of the patients blinded to the CGMS data; IADPSG—International Association of Diabetes in Pregnancy Study Groups; WG—weight gain.

## Data Availability

Data sharing is not applicable to this article.

## Abbreviations

The following abbreviations are used in this manuscript:

ADRR	Average daily risk range
AUC	Area under the curve
BGRI	Blood glucose risk index
BMI	Body mass index
CG	Control group
CGMS	Continuous glucose monitoring systems
CI	Confidence interval
DM	Diabetes mellitus
GDM	Gestational diabetes mellitus
GW	Gestational weeks
HbA1c	A1c fraction of the haemoglobin
HP	Hyperglycaemia patterns
HSSV	Hours per day spent in severe variability
LAGE	Largest amplitude of glycaemic excursions
LGA	Large for gestational age
MAD%	Percentage of mean absolute differences
MAGE	Mean amplitude of glycaemic excursions
MAGE2	MAGE value in the 5th week
MBG	Mean blood glucose
MODD	Mean of daily differences
NICU	Neonatal intensive care unit
NS	Not specified
OGTT	Oral glucose tolerance test
OR	Odds ratio
PIH	Pregnancy induced hypertension
PPH	Postpartum haemorrhage
PT	Pharmacological treatment
PTB	Preterm birth
PTL	Preterm labour
RDS	Respiratory distress syndrome
RT-CGMS	Real-time CGMS
SD	Standard deviation
SDBG	SD of blood glucose
Se	Sensitivity
Sp	Specificity
SMBG	Self-monitoring blood glucose
T1DM	Type 1 DM
T2DM	Type 2 DM
TAR	Time above range
TBR	Time below range
TIH	Time in hyperglycaemia
